# Machine learning-assisted stiffness prediction in high-cell-density bioprinting

**DOI:** 10.1631/bdm.2400454

**Published:** 2025-04-24

**Authors:** Jiaao Guan, Yazhi Sun, Emmie J. Yao, Yi Xiang, Mary K. Melarkey, Grace Y. Lu, Amelia H. Burns, Nancy Zhang, Shaochen Chen

**Affiliations:** 1 Aiiso Yufeng Li Family Department of Chemical and Nano Engineering, University of California San Diego, La Jolla, CA 92093, USA; 2 School of Biological Sciences, University of California San Diego, La Jolla, CA 92093, USA; 3 Department of Biology and Biological Engineering, California Institute of Technology, Pasadena, CA 91125, USA; 4 Shu Chien-Gene Lay Department of Bioengineering, University of California San Diego, La Jolla, CA 92093, USA

**Keywords:** Bioprinting, Stiffness, Machine learning, High cell density, Tissue engineering

## Abstract

Bioprinting of cell-laden hydrogels is a rapidly growing field in tissue engineering. The advent of digital light processing (DLP) three-dimensional (3D) bioprinting technique has revolutionized the fabrication of complex 3D structures. By adjusting light exposure, it becomes possible to control the mechanical properties of the structure, a critical factor in modulating cell activities. To better mimic cell densities in real tissues, recent progress has been made in achieving high-cell-density (HCD) printing with high resolution. However, regulating the stiffness in HCD constructs remains challenging. The large volume of cells greatly affects the light-based DLP bioprinting by causing light absorption, reflection, and scattering. Here, we introduce a neural network-based machine learning technique to predict the stiffness of cell-laden hydrogel scaffolds. Using comprehensive mechanical testing data from 3D bioprinted samples, the model was trained to deliver accurate predictions. To address the demand of working with precious and costly cell types, we employed various methods to ensure the generalizability of the model, even with limited datasets. We demonstrated a transfer learning method to achieve good performance for a precious cell type with a reduced amount of data. The chosen method outperformed many other machine learning techniques, offering a reliable and efficient solution for stiffness prediction in cell-laden scaffolds. This breakthrough paves the way for the next generation of precision bioprinting and more customized tissue engineering.

## Graphical Abstract

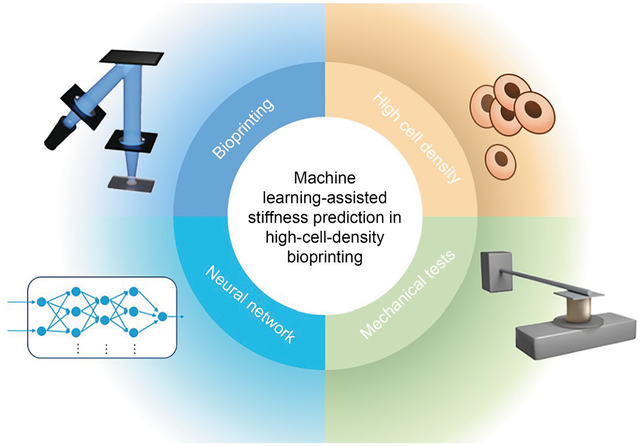

## 1 Introduction

The high demand for creating functional tissues to study biological mechanisms or replace damaged tissues has led to the development of various techniques for the fabrication of artificial tissues. Among these, three-dimensional (3D) bioprinting has attracted considerable attention in recent years owing to its ability to deposit various types of biomaterials and cells with high spatial resolution in a controlled manner [1, 2]. Additionally, advancements in four-dimensional (4D) printing enhance the functionality of fabricated structures, enabling them to respond to external stimuli [3].

Within 3D bioprinting, digital light processing (DLP) 3D printing possesses the advantage of efficiently fabricating complex 3D structures. This technique uses a continuous light projection onto the sample stage to crosslink the bioink precursor in a plane-by-plane manner. In comparison to inkjet or extrusion-based 3D printers, which allow only dot-by-dot or line-by-line fabrication [4, 5], DLP offers significantly faster and more biocompatible fabrication. Furthermore, its rapid fabrication supports high cell viability and allows for versatile bioink formulations. By contrast, extrusion-based printing requires a specific range of bioink viscosity to function properly. Adding cells would either hinder the printability or risk cell viability owing to the large shear stress. A larger nozzle size is often used to mitigate the shear stress to ensure the cell viability while trading off the printing quality. DLP printing relies less on the rheological properties of the bioink, granting greater flexibility in bioink compositions. This enables the fabrication of artificial tissues at arbitrary cell densities.

Bioprinted tissues possessing tissue functions with complex structures, such as the liver [6] and neural [7] tissues, have been achieved through the DLP bioprinting technique. These tissues have demonstrated essential functions including vascularization, nutrient transport, and cell-specific responses, significantly advancing their potential for clinical and therapeutic applications. Additionally, the versatility of 3D printing has enabled the fabrication of complex auxetic structures with a negative Poisson’s ratio [8]. These structures have been utilized as substrates to investigate cellular responses to mechanical forces [9, 10]. Despite these advances, the cell density in fabricated tissues remains a key limitation. Current bioprinted tissues typically achieve cell densities of only a few million cells per mL. This is around 1% of the cell density in actual tissues [11]. This low cell density in the bioprinted tissues often limits cell–cell interactions and often fails to replicate the physiological properties observed in vivo.

However, when printing high-cell-density (HCD) samples using DLP bioprinters, light scattering caused by the presence of cells interferes with both light intensity and printing resolution. Recently, this issue was alleviated by including a biocompatible supplement, iodixanol (IDX), in the bioink. Matching the refractive index of the prepolymer solution to that of the cytoplasm allowed for a resolution of 50 μm for 1×10^8^ cells/mL. Following a 14-d perfusion culture at 4×10^7^ cells/mL cell density prints, 66% of the cells were viable [12]. DLP bioprinting uses photocrosslinkable polymer precursors that are cured under light exposure through free-radical polymerization. Factors such as light intensity, exposure time, and the concentrations of both precursor solutions and photoinitiators significantly affect the stiffness of the printed structures [4, 13]. Although there have been efforts to improve printing fidelity, the impact of cells on the mechanical properties of the extracellular matrices (ECMs) remained underexplored.

The stiffness of ECMs is critical in influencing various biological processes, including impacting cell phenotype and behavior [14], making precise control essential in bioprinted tissues. Crosslinking density is a crucial factor that determines the mechanical stiffness of the photocrosslinkable hydrogels. These hydrogels undergo a free-radical chain growth reaction mechanism, including initiation, propagation, and termination. The extent of photocrosslinking is influenced by many factors, including the bioink composition, such as the concentrations of precursor monomers and photoinitiators, as well as printing parameters like light power and exposure time. These variables collectively affect the concentration of free radicals [4]. Prolonging the exposure time is often the most effective way to enhance stiffness than adjusting other parameters. For example, increasing light power can damage cells (e.g., ultraviolet-induced damage), raising photoinitiator concentrations increases cytotoxicity, and higher monomer concentrations may hinder nutrient diffusion through the hydrogel matrix. Moreover, in HCD printing, where cells comprise a large portion of the bioink, adjusting the bioink composition has a more limited effect compared to traditional low-cell-density printing.

The high volume ratio of cells in HCD bioinks significantly alters their optical properties compared to those of acellular biopolymer precursors. To better match the differences in the refractive index of different cells owing to their organelle composition [15], the IDX concentration needs to be optimized to match the refractive index of the cytoplasm and that of the prepolymer solution depending on the specific cell type [12]. While incorporating IDX can mitigate cell-induced light interruptions, including absorption, reflection, and scattering, these interruptions could not be fully eliminated. Consequently, the bioink composition and the optimal effect of IDX inclusion should be optimized individually for each cell type and density. This leads to different printing results when applying established printing parameters to different bioinks, even when the same base prepolymer is used.

Measuring stiffness for each cell density and type under comprehensive printing conditions is both time-consuming and costly, even for cell types that are readily available. This challenge becomes more pronounced when dealing with precious cells, such as primary cells isolated from tissues. These cells are often available in limited quantities, exhibit donor-to-donor variability, and are generally insufficient to support detailed stiffness measurements under various bioprinting conditions. Therefore, to obtain printing parameters for the expected stiffness, there is a pressing need to predict a wide range of stiffness based on printing parameters. Alternatively, such models should be able to determine the necessary printing parameters to achieve specific stiffness while relying on minimal input data and minimal use of costly cells or materials.

Machine learning (ML) is a powerful computational technique that can learn the underlying patterns and relationships from data without specifically requiring explicit knowledge of the physics involved. ML has gained significant traction in various 3D printing and bioprinting applications, including quality control, defect detection, design optimization, and material property prediction [16–21]. In terms of mechanical property prediction in bioprinting, Goh et al. developed an ML model to predict the shore hardness and compressive modulus of a multimaterial tissue-mimicking anatomical model fabricated using material jetting 3D printing. They successfully applied the trained model to optimize design parameters [22]. Omigbodun et al. utilized ML algorithms to predict the mechanical properties of polylactic acid and calcium hydroxyapatite scaffolds made by fused deposition modeling [23]. Kiratitanaporn et al. applied ML to predict the poly(glycerol sebacate) acrylate scaffold stiffness fabricated with DLP-based and two-photon polymerization-based 3D printing [24]. Despite these advances, very few studies have focused on applying ML to analyze the mechanical properties of bioprinted scaffolds. Notably, there are currently no reports specifically addressing ML-based prediction and modulation of scaffold stiffness in HCD bioprinting. Given the importance of the high cell density for creating functional tissues and organs, our work contributes to analyzing and predicting mechanical properties using ML in HCD cell-laden bioprinting. By training these models with ML algorithms, we aim to identify optimal bioprinting parameters more efficiently, minimizing costly trial-and-error approaches.

A challenge of applying ML to HCD bioprinting is the difficulty in collecting real printed sample measurements owing to the high cost and long processing time required for both cell culturing and the bioprinting of HCD biomaterials. Therefore, we consider utilizing a transfer learning technique. This approach allows a pretrained model developed for one cell type to enhance the training for a new cell type, thereby reducing the amount of required data. Transfer learning has traditionally been employed in domains with large public datasets and accessible pretrained models. For example, Shin et al. applied transfer learning from large-scale nonmedical image datasets to medical imaging tasks with limited data availability [25]. Despite the difference between the datasets, their findings highlighted that medical image recognition tasks could benefit from pretrained models on natural images. This method has been extended to image-based defect detection tasks in different 3D printing techniques such as fused deposition modeling [26], selective laser sintering [27], direct energy deposition [28], and DLP [29]. However, for material property predictions where nonimage data serve as input features, public datasets for pretraining are often unavailable. Instead, transfer learning in such cases relies on transferring knowledge from one system to another using unique experimental or simulation data. Thomas et al. demonstrated the use of physics-guided transfer learning to predict the thermal conductivity of short fibers printed with extrusion deposition 3D printing techniques [30]. They successfully transferred knowledge from one dataset to improve learning in a different 3D printing system that would induce different microstructures when printing the same material. Pashmforoush and Seyedzavvar applied transfer learning using artificial neural networks (NNs) to efficiently learn to predict the mechanical properties of different material types fabricated using selective laser melting [31]. They transferred knowledge trained from 321 source samples to enhance the learning of 35 samples of a different metal, relying entirely on previously published data. Our study adopts a similar methodology by leveraging transfer learning to enhance scaffold stiffness predictions for one cell type using knowledge gained from a pretrained model based on another cell type. It is worth noting that all our data were collected through real bioprinting experiments with living cells and consecutive mechanical testing. Our study serves as additional evidence supporting the effectiveness of transfer learning in small data scenarios that are common in bioprinting. Our application of transfer learning is novel in terms of using only real bioprinting data without any simulation or synthetic data. We are also the first to study cell-encapsulated HCD bioprinting stiffness using ML and transfer learning.

**Fig. 1:**
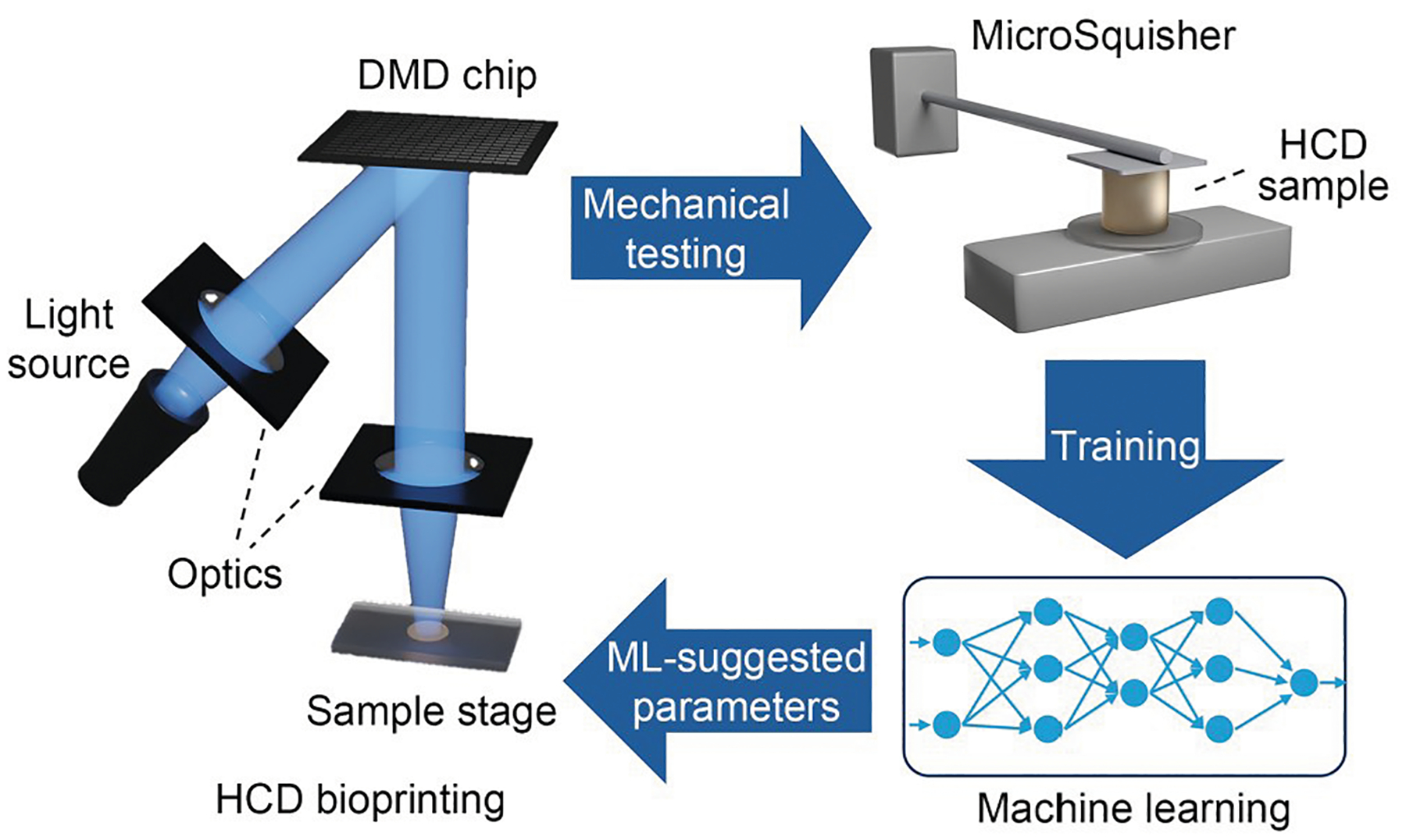
Schematic of ML-assisted HCD bioprinting. Initially, HCD samples containing one cell type were printed under various light exposure conditions. Mechanical tests were conducted to measure the stiffness, and the resulting data were used to train the ML algorithms. The trained model then predicted the overall stiffness distribution for different cell types based on the limited input parameters from the new cell type. The ML-suggested parameters were subsequently applied to assist HCD bioprinting under new conditions. DMD: digital micromirror device

In this study, we aim to predict and control the scaffold stiffness in HCD bioprinting with ML methods. We employed an NN ML model to predict scaffold stiffness in HCD bioprinting based on cell density and light exposure and applied transfer learning to extend the model to a new cell type (Fig. 1). Photocrosslinkable polymers derived from natural materials are commonly used in tissue engineering owing to their biocompatibility and biodegradability with mammalian cells when compared to synthetic polymers. For this reason, we chose methacrylated gelatin (GelMA) as our base prepolymer. To study how cells affect the bioprinted sample stiffness, we kept all other material compositions consistent across experiments, including the concentrations of GelMA, photoinitiator, and the optimal IDX concentration for each cell type. To simplify control over light exposure, a fixed light intensity of 32.54 mW/cm^2^ was used with variations in exposure duration. Initially, we printed samples using a low-cost 293T cell line, spanning a comprehensive range of cell densities and light exposure times, to generate stiffness data. The resulting data were then used to train the NN model with input variables, including cell density, exposure time, and stiffness measurements. Transfer learning was subsequently applied to predict the stiffness of a more precious model cell, the hepatoma HepG2 cell line, using a reduced dataset. Compared with 293T epithelial-like cells, HepG2 cells have a larger size and a different refractive index. This workflow exemplifies how the trained model can be adapted to predict stiffness distributions for cell types that are more precious and limited. Our model demonstrated an excellent fit with the stiffness data derived from 293T cells and effectively predicted the stiffness of HepG2 cells using limited input data. These results indicated the model’s potential to be further applied in scaling up DLP bioprinting conditions to achieve ideal stiffness in broader applications.

## 2 Materials and methods

### 2.1 HCD bioprinting

#### 2.1.1 Acellular bioink preparation

GelMA was synthesized using gelatin from porcine skin (gel strength 300, Type A, Sigma Aldrich, St. Louis, MO, USA). Briefly, gelatin was dissolved at 10% (0.1 g/mL) in a 3:7 carbonate–bicarbonate buffer (pH=9) at 50 °C. Once fully dissolved, methacrylic anhydride (MA; Sigma Aldrich, St. Louis, MO, USA) was added to the gelatin solution at 0.085 mL/g to achieve 85% methacrylation. This mixture was then stirred for 1 h at 50 °C. The solution was transferred into dialysis tubing (Cat. #888–11539, 14 kDa cutoff, Spectrum Laboratories, USA) to remove the unreacted MA groups. Dialysis was performed against distilled water for one week at 45 °C. After dialysis, the GelMA solution was frozen overnight at −80 °C and lyophilized in a freeze dryer (Labconco, USA). A stock GelMA solution (20% (0.2 g/mL)) was prepared by dissolving freeze-dried GelMA in Dulbecco’s phosphate-buffered saline (DPBS, Gibco, USA). Lithium phenyl-2,4,6-trimethylbenzoylphosphinate (LAP; TCI America, USA) was dissolved in DPBS to create a 4% (0.04 g/mL) stock solution, which was sterilized using 0.22 μm filters. An IDX (60% (0.6 g/mL)) solution (OptiPrep^™^ density gradient medium) was purchased from Sigma-Aldrich. The acellular bioink was prepared by combining the stock solutions of GelMA and LAP to a final concentration of 5% (0.05 g/mL) and 0.4% (4 mg/mL), respectively, in DPBS. The IDX concentration was optimized for each cell type as previously described [12]. The final IDX concentration was 30% (0.3 g/mL) for 293T cells and 20% (0.2 g/mL) for HepG2 cells.

#### 2.1.2 Cell culture

293T (ATCC, USA) cells were cultured in Dulbecco’s modified Eagle’s medium (ATCC, USA) supplemented with 2 mmol/L L-Glutamine (Cat.#25030081, Gibco), 10% fetal bovine serum (Cat. #SH30396.03, Cytiva, USA), and 1% penicillin/streptomycin (Cat.#15140122, ThermoFisher, USA). HepG2 (ATCC, USA) cells were cultured in Eagle’s minimum essential medium (ATCC, USA) supplemented with 10% fetal bovine serum and 1% penicillin/streptomycin. Both cell types were thawed at 1×10^6^ cells/mL and cultured in a 75 cm^2^ flask (Corning, USA). The medium was refreshed every 3 d. Cells were subcultured when the flask reached 80% confluency, following the manufacturer’s instructions.

#### 2.1.3 3D bioprinting process

To prepare the cellular bioink, 293T and HepG2 cells were digested with 0.25% trypsin (Cat.#25200–056, Gibco). The cells were then aliquoted to predetermined cell densities and spun at 200*g* for 5 min prior to printing. The medium was then removed, and 10 μL of acellular bioink material was mixed with the cell pellet. The mixture was gently pipetted to achieve a uniform cell suspension. For mechanical measurements, samples were prepared using a setup consisting of a polydimethylsiloxane (PDMS) sheet and a methacrylated coverslip, separated by PDMS spacers with a thickness of 500 μm, all placed on top of a glass slide. The bioink was injected into the gap between the PDMS sheet and the methacrylated coverslip. Then the glass slide was placed on the sample stage of an in-house DLP bioprinter.

The DLP printer utilized a digital micromirror device (DMD) containing an array of millions of micromirrors, each of which could be individually turned on or off by inputting a two-dimensional digital mask. The DMD device reflected light from a source with a wavelength of 365 nm, projecting the patterned light beam passing through the optics and being focused on the bioink reservoir between the PDMS sheet and the methacrylated coverslip. Each sample was exposed to a circular light pattern with a diameter of 500 μm under a light intensity of 32.54 mW/cm^2^ for a predetermined duration.

#### 2.1.4 Mechanical testing

Mechanical measurements were conducted on the day of printing. Cylinder samples with a thickness of 500 μm and a diameter of 500 μm were printed. Compression tests were carried out using a MicroTester (CellScale, USA) machine. In brief, each sample was placed beneath a cantilever plate and compressed to a displacement of 90 μm at a speed of 6 μm/s before returning to its original shape to complete one compression cycle. Force and displacement data were recorded by the MicroTester for each cycle. The first two cycles removed the hysteresis caused by internal friction. The compressive modulus was calculated from data of the third cycle using customized MATLAB scripts.

#### 2.1.5 Cell viability test

Live/dead staining was conducted on samples with a cell density of 2×10^8^ cells/mL (Figs. S1 and S2 in the supplementary information). A detailed viability study on HCD scaffolds produced through DLP printing using identical material compositions was available in prior work [12].

### 2.2 Computational methods

#### 2.2.1 Data selection

To investigate the relationship among mechanical properties, cells, materials, and 3D printing parameters, sufficient stiffness data were collected from mechanical tests on samples printed with varying parameters. Cell density within the material and light exposure during bioprinting were chosen as the variable parameters. For the initial data collection on 293T test samples, cell densities of 0, 5×10^7^, 1×10^8^, and 2×10^8^ cells/mL were tested under light exposure durations of 8, 14, 20, 30, and 40 s for a total of 20 different conditions. Two additional conditions under 2.5× 10^7^ cells/mL with 20 s exposure and 40 s exposure were later added to improve confidence in regions with high measurement variance. This brought the total to 22 distinct conditions, with 3–8 replicates per condition (*n*⩾3). For HepG2 data collection, 15 conditions were randomly selected from the 22 previous parameter combinations, with 2–7 replicates per condition (*n*⩾2). It is important to note that the total measurements collected for HepG2 were fewer than half of the measurements taken for 293T, simulating scenarios with limited data availability.

#### 2.2.2 ML

ML methods are robust self-learning computer algorithms that can perform classification or regression tasks given sufficient training data. A growing number of ML techniques have been used in material research, such as material property prediction [24, 32, 33]. Given the high costs, labor-intensive efforts, and time-consuming processes involved in mechanically characterizing 3D bioprinted samples, we aimed to utilize the ML technique to precisely predict the sample stiffness of any given cell-encapsulated 3D-printed scaffold with varying printing parameters and cell densities.

The ML method employed was artificial NNs, also known as multilayer perceptron or deep NNs [34, 35]. NNs are a powerful ML approach, renowned for their outstanding performance as universal function approximators that can model complex, nonlinear functions provided that an adequate dataset is available [36]. Considering the relatively low dimensionality of the input and output spaces, we constructed a three-layer fully connected NN model for our task. Each layer consisted of multiple hidden neurons (Eq. (1)), where each *j*th neuron *z*_*j*_ was composed of a weighted sum of the input features *x*_*i*_ (*i* = 1, 2, …, *N*) of this layer, plus a scalar bias *b*_*j*_, and then passed through a rectified linear unit (ReLU) [37] denoted as *f* (·). The output of one layer served as the input of the next layer, with the initial input features being cell density and exposure time, and the final output representing the predicted stiffness.


(1) $$z_{j}=f\left(b_{j}+\sum_{i=1}^{N}x_{i}\;w_{ij}\right).$$

**Fig. 2:**
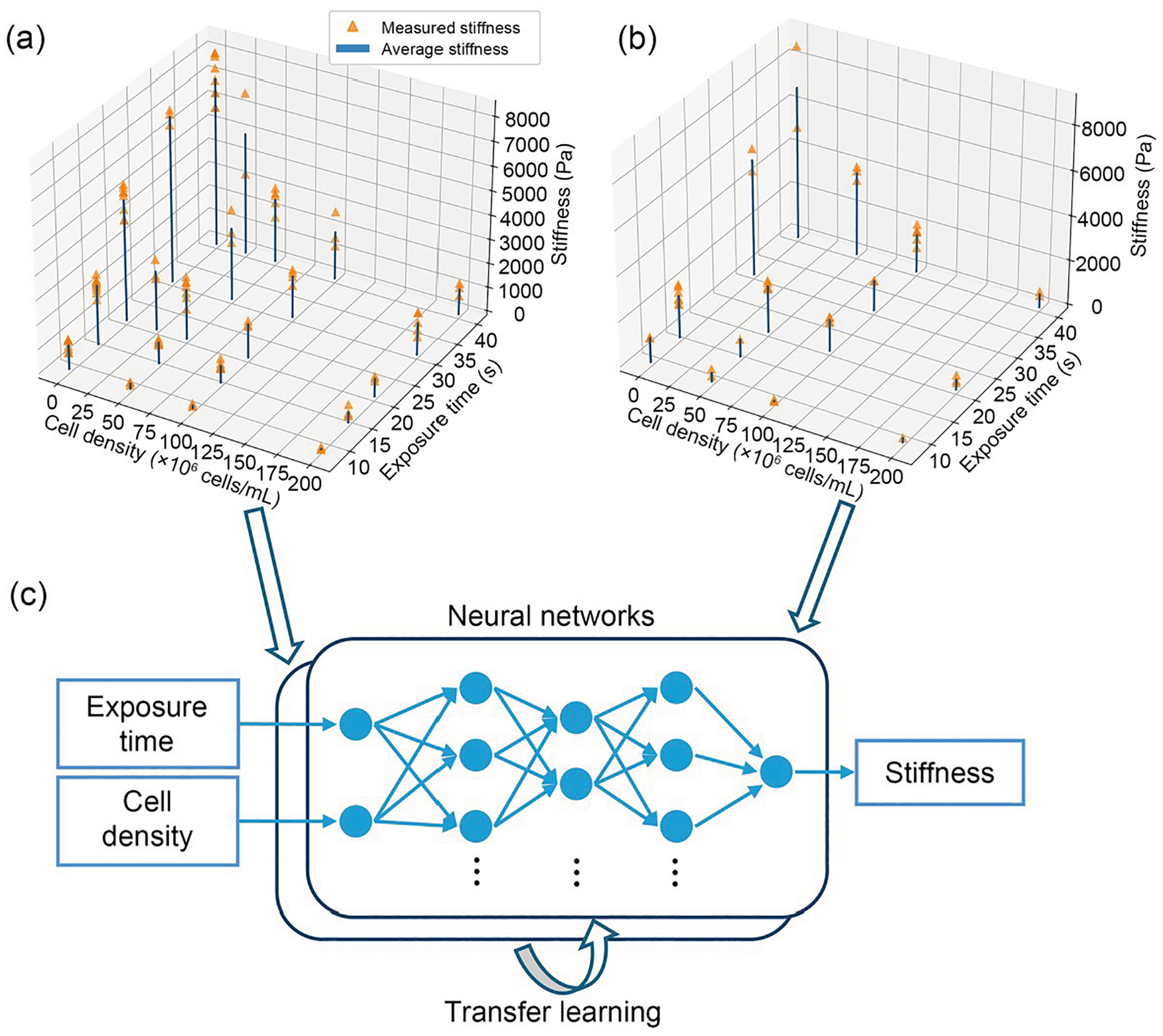
Mechanical testing results and ML schematics. Mechanical tests resulted in compressive stiffness values for 3D-printed samples with 293T cells (a) and HepG2 cells (b) in the bioink. The individual stiffness measurements are shown as orange triangular dots. The average stiffness values for each condition are shown as blue bars. (c) NN architecture and transfer learning schematics

Both the weight *w*_*ij*_ for the weighted sum and the bias *b*_*j*_ were trainable parameters for the NN model. The number of neurons was carefully selected as 16, 4, and 8 for the three layers to prevent overfitting, as explained in the subsequent sections (Fig. 2c). The NN model training method was a gradient-descent-based backpropagation method called Adam, with the mean absolute error (MAE) loss chosen as the optimization criterion [38]. Kaiming initialization was applied to initialize the model weights efficiently [39].

To benchmark the NN model, five other simple and commonly used ML methods were implemented for comparison. These included the linear least square regressor, linear quadratic regressor [40], support vector machine (SVM) regressor [41], random forest (RF) regressor [42], and a single-layer four-neuron NN model.

In addition, transfer learning was employed to train the NN using HepG2 cell data based on a previously trained model with 293T cell data (Fig. 2c). Transfer learning is a method that retrains an existing model to adapt to a different dataset [43, 44]. It is commonly applied to similar datasets where certain datasets have relatively limited data. The process involves first training the model on a dataset with a sufficient volume of data, followed by fine-tuning it on a smaller, less comprehensive dataset. There are three typical methods of transfer learning for NN models. The first involves fine-tuning the entire model, the second freezes the early layers while fine-tuning only the final or few layers, and the third freezes the whole model while attaching and training an additional layer to the end of the model. For large models, the latter two approaches are often preferred due to their efficiency, as they require retraining only a small portion of the model. In our case, our NN model was relatively small, with only three layers. Thus, the first approach, fine-tuning the whole model, was utilized for transfer learning.

#### 2.2.3 Model overfitting

NN models are prone to overfitting. Overfitting occurs in ML algorithms when a model becomes overly tailored to the training data, losing its ability to generalize and performing poorly on unseen data [34]. A common way to prevent overfitting is to allocate a portion of the experimental data as a validation set, separate from the training set. These validation data are used during training to halt the process when the model’s performance begins to deteriorate on the validation set. As previously emphasized, cell-incorporated bioprinting is very costly and time-consuming; thus, separating the validation data from the training data was not feasible. Instead of terminating the model training process based on the validation set performance, we employed a specific hyperparameter tuning technique to predetermine the optimal number of training epochs and learning rate, ensuring that the model would cease training at an appropriate point to maintain performance and mitigate overfitting. Furthermore, L2 regularization was applied to the model weights. This technique helped constrain the model by discouraging extreme weight values, reducing the likelihood of overfitting. In addition, we implemented dropout on the hidden layers of the NN model and compared it with a model without dropout. Dropout regularization randomly zeroed out the weight of hidden neurons at a given probability, which promotes even importance across neuron weights and thus helps prevent model biasing and overfitting [35, 45].

#### 2.2.4 Evaluation metrics

We chose MAE, root mean square error (RMSE), mean absolute percentage error (MAPE), and the coefficient of determination (*R*^2^) as our evaluation metrics. These metrics were used to measure the differences or scores between the model predictions and the ground truth stiffness measurements to assess the performance of each model. MAE was chosen as the primary evaluation metric because it has the advantage of being directly interpretable as the average prediction error in the same unit as the stiffness values. Furthermore, MAE aligns with the loss function during the NN model training.

Typical ML evaluation processes separate a portion of the data away from the model training and apply the evaluation metrics on that standalone evaluation set. However, unlike typical big-data-based ML, in bioprinting, the high costs and lengthy experimental processing times limit the amount of data available. Therefore, every data point is precious and contributes to the quality of the resulting NN model. To properly evaluate the NN model, we first applied the MAE metric to the model trained on the complete dataset and compared its predictions against the training data to obtain training errors or training losses. To further assess the model’s ability to predict stiffness for previously unseen printing conditions, we implemented leave-one-out (LOO) cross-validation [46, 47]. The LOO cross-validation is a special case of the *k*-fold cross-validation. The *k*-fold cross-validation evenly separates the available dataset into a predetermined number of subsets. At every iteration, we train a model on the dataset by removing one of the subsets and then test the trained model on the removed subset. In this way, the model is always being tested on a non-training set, and after repeating for all *k*-folds, the averaged testing score is a reasonable quality assessment for the model on this dataset. At each iteration of the *k*-fold cross-validation, the removed subset of data used to evaluate the model is unseen by this model during the training process. Therefore, the *k*-fold cross-validation result accounts for the model’s generalizability to unseen data in the given data distribution. LOO cross-validation is a special variant of *k*-fold cross-validation where the number of folds equals the total number of data points. This means that, in each iteration, only one data point is left out. The LOO cross-validation is known to be unbiased and is suitable for small datasets. All four evaluation metrics were applied with LOO cross-validation.

## 3 Results

### 3.1 Stiffness data collection

To generate the stiffness data for training, 3D-printed samples composed of the 293T cell line were selected owing to the cells’ rapid proliferation and low maintenance costs. In addition, the HepG2 cell line was used to replicate the common bioprinting situation where we had limited cells to generate experimental data. Compression mechanical tests were then conducted to obtain the stiffness of the printed samples (Fig. 1). Two primary variables, cell density and light exposure time, were varied during the printing process. It is worth noting that many factors can affect the degree of photocrosslinking in DLP printing, such as light intensity, photoinitiator concentration, and polymer precursor concentration. These factors affect photocrosslinking density by adjusting free-radical concentrations. To simplify the experimental setup, a constant light intensity was applied across all samples. The other variables were fixed for consistency, namely the GelMA and photoinitiator concentrations. The IDX concentration was optimized for each cell type, set at 30% IDX for 293T cells and 20% IDX for HepG2 cells. A total of 104 samples were tested using 293T cells under 22 different printing conditions, and 46 samples were tested with HepG2 cells under 15 unique printing conditions.

As shown in Figs. 2a and 2b, at a constant cell density, longer light exposure times produced stiffer samples. These results align with our intuition: prolonged light exposure generates more free radicals to polymerize the precursor solution, forming a denser network, whereas a higher cell density increases light scattering, thus reducing polymerization efficiency. For training the ML model, the stiffness values were averaged for each printing condition, and each condition was treated as an individual data point.

### 3.2 Hyperparameter tuning and architecture searching

Before training our NN model with the collected data, we first determined the appropriate model architecture and training hyperparameters. The goal of this process was not only to train a fitting model but also to prevent overfitting, or in other words to maximize the model’s generalizability to unseen data.

For the NN model, the architecture includes the number of layers, the number of neurons per layer, and the activation functions. In our case, we decided to use the widely used and robust ReLU function for all layers. Therefore, we only needed to decide the number of layers and the number of neurons per layer for the NN architecture search. The goal was to design a model large enough to capture the underlying trends of the data while keeping it compact enough to reduce the risk of overfitting.

The training hyperparameters that were tuned included the learning rate, the number of epochs, and the dropout rate. Again, we decided that the tuning goal would be to further reduce model overfitting. We set the exponential decay rate coefficients for the Adam optimizer at 0.9 and 0.999, following the original Adam paper [38], and we set the weight decay factor at 10^−5^ for the L2 regularization of the model weights. Since the dataset was relatively small, each training epoch was performed using the entire batch.

In the early stages of modeling, our fitted 293T training model was overfitted. We started with a relatively high initial learning rate of 0.1, relying on the learning rate annealing effect of the Adam algorithm to gradually adjust it during training. We trained our initial model all the way until convergence, which we defined as the point at which the training loss was not improving for 5000 epochs. By manually testing a few NN model architectures, the initial model was chosen to be a three-layer NN with 16 neurons on each layer, which could consistently fit the training data to an MAE loss of less than 10 at convergence, where the MAE loss value of 10 could be seen as the average 10 Pa stiffness error. Given the material stiffness range of several hundred Pa to a few thousand Pa, this error was deemed negligible.

**Fig. 3:**
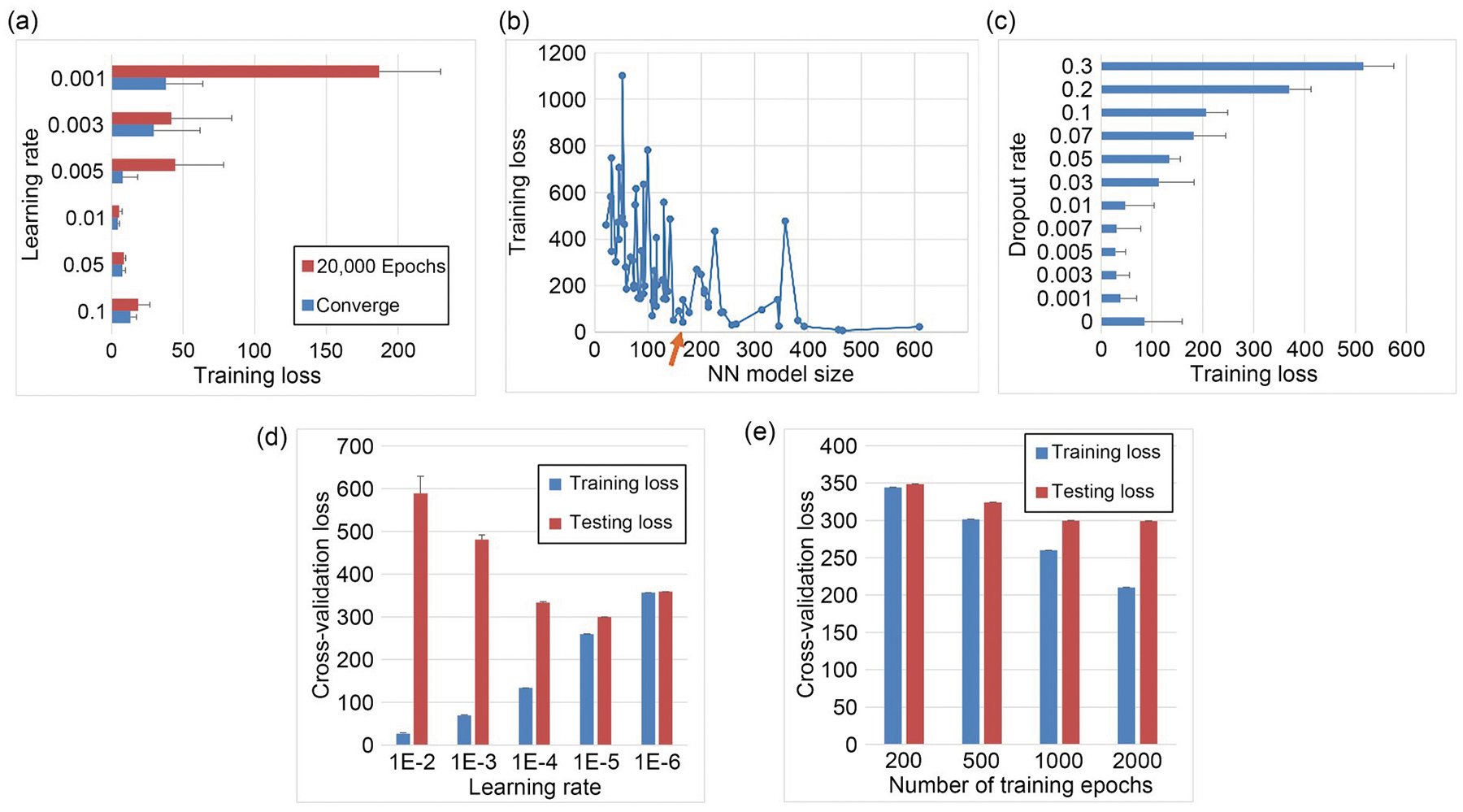
NN hyperparameter tuning and architecture searching. (a) Average training MAE losses for different learning rates with the largest NN model in the range of architectures. Each training process was either stopped early at 20,000 epochs or trained until convergence. (b) Average training MAE losses for different NN model architectures represented by the model size within the searching range. The orange arrow indicates the chosen model architecture. (c) Average training MAE losses for the optimal model with different dropout rates. (d) LOO cross-validation MAE losses for the model trained with the transfer learning method under different learning rates for 1000 epochs. (e) LOO cross-validation MAE losses for the model trained with the transfer learning method under 1E-5 learning rate for various numbers of epochs. Models in (a–c) were trained on 293T data, and models in (d) and (e) were trained on HepG2 data using transfer learning. Data are expressed as mean±standard deviation (*n*=10)

After we decided on the initial model, we optimized the learning rate. Using the complete 293T dataset, we trained the model on different learning rates ranging from 0.1 to 0.001, repeating each experiment 10 times to account for variability due to random initialization. The result showed that the 0.01 rate consistently produced the minimum training loss at convergence (Fig. 3a). We also experimented with stopping the training at a fixed 20,000 epochs instead of waiting for full convergence. While this approach yielded similar results, smaller learning rates performed worse as the training terminated prematurely.

Once the optimal learning rate was determined, we proceeded to search for the optimal NN architecture. Using our consistently converging initial architecture, we tested a range of smaller architectures. Specifically, we examined three-layer NNs with each layer having 2, 4, 8, or 16 neurons, where the binary number choices were the typical computer science convention to utilize computer memory usage. After plotting model size against training loss, we selected a three-layer NN with 16, 4, and 8 neurons per layer, striking a balance between low average training loss and compact model size (Fig. 3b). It is important to note that each architecture was tested five times, and the standard deviation among the training losses corresponded closely to the overall trend. Thus, it was not included in the plot. The resulting 16-4-8 NN model was named full NN or three-layer full NN in the following context as different from the simple one-layer NN as a comparison ML model.

Next, we tuned the dropout rate. Dropout is an effective technique for reducing overfitting and model bias by evening out the significance of individual weights and stabilizing the training process. This helps the model avoid becoming trapped in local optima. Testing various dropout rates with 10 repetitions for each condition revealed that a dropout rate of 0.005 yielded the most consistent convergence and the lowest loss (Fig. 3c).

Without a standalone validation set, we lacked strong evidence to establish an early stopping condition. Therefore, we maintained our established convention of stopping training when the model showed no improvement for 5000 consecutive epochs. With the chosen hyperparameters, model training typically converged within 20,000 epochs (Fig. S3 in the supplementary information).

By tuning the above hyperparameters and NN architecture, we ensured that the NN trained with the 293T cell data maintained high quality. To further prove that we could use the same model and apply transfer learning with a small dataset and still obtain a model with accurate stiffness prediction results, we applied this strategy to our HepG2 data. Transfer learning relies on the similarity between the two datasets and typically requires fewer epochs and adjustments to the learning rate as the base model already had knowledge of a similar dataset. We further adjusted the learning rate specifically for transfer learning, and we applied LOO cross-validation owing to the faster training process of transfer learning. By evaluating various learning rates, we found that although the training loss is worse with lower learning rates, the testing loss is better (Fig. 3d). Balancing these two outcomes, we selected a learning rate of 10^−5^. With this learning rate, we further explored different training epochs and found that the testing loss no longer improved after 1000 epochs; therefore, we adopted this value as our endpoint (Fig. 3e).

### 3.3 Model training and evaluation

**Fig. 4:**
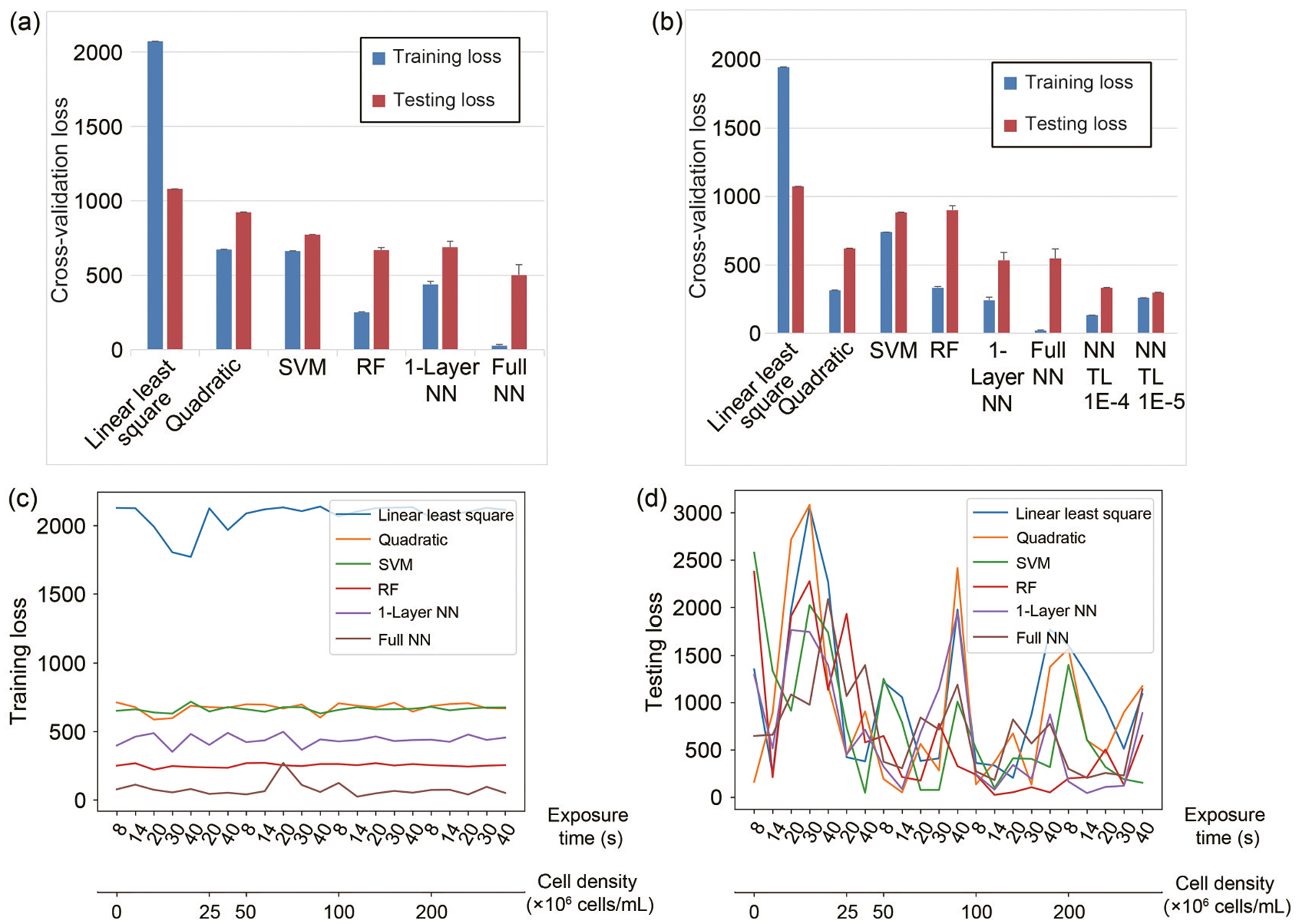
ML result evaluation and comparison. (a) LOO cross-validation losses on the 293T data for different ML methods, including linear regression, quadratic regression, SVM regression, RF regression, 1-layer NN, and three-layer fully-connected NN. (b) LOO cross-validation losses on the HepG2 data for different ML methods, including the six methods in (a) as well as two transfer learning (TL) methods based on a previously trained three-layer fully-connected NN with learning rates of 1E-4 and 1E-5. (c) Training losses for LOO cross-validation, where the *x*-axis shows the left-out data points for each fold. (d) Testing losses for LOO cross-validation, where the *x*-axis shows the left-out data points for each fold. Data in (a, b) are expressed as mean±standard deviation (*n*=10)

With the hyperparameters selected earlier, we fully trained our model, evaluated it, and compared it with other ML methods. The evaluation was conducted using LOO cross-validation, where the mean and standard deviation of the LOO cross-validation errors were calculated across 10 repeated model training iterations for each approach. Figure S3 (supplementary information) shows an example of the converging MAE training loss plot during the training process of the three-layer full NN. After comparing the LOO cross-validation results for all ML models trained with the 293T dataset, we found that the full NN consistently outperformed the others, achieving the lowest training MAE loss and the lowest testing MAE loss (Fig. 4a). Similar conclusions were drawn from the average testing errors and scores of additional evaluation metrics (Table 1). Further investigating the details of the LOO cross-validation results, we attempted to investigate the individual iterations within the LOO cross-validation. Figures 4c and 4d respectively show the individual training losses and testing losses during each LOO iteration, with the *x*-axis indicating the removed or tested condition for that iteration. We observed that although all iterations exhibited similar training losses, there were several iterations with extra high testing losses, meaning that those conditions contained critical information that could hardly be derived from the other conditions. Those critical conditions were mainly noticed among iterations involving 0 cells/mL cell density and 40 s exposure time for the full NN. These edge conditions proved to be the most crucial data points, an observation that could guide future data collection efforts if this study is extended to other cell types. Excluding these edge conditions significantly reduced the average MAE to below 300 Pa, an exceptional error given that the standard deviation of the stiffness measurements was also around 300 Pa.

While our trained NN model demonstrated reasonable stiffness prediction despite being developed with a strictly limited dataset, it is important to emphasize that its predictive accuracy would likely improve with the inclusion of more data. Our efforts were vastly oriented toward improving model generalizability, ensuring it can effectively predict both seen and unseen data using the available information. Next, we will show how we could even extend this method to improve the learning of another cell type with even fewer data available.

### 3.4 Transfer learning

Transfer learning utilizes the information from a previously trained base model and adapts it to a new dataset. We selected HepG2 cells as the model for applying transfer learning owing to their larger cell size and different scattering properties. Leveraging the previously trained full NN model based on the 293T dataset, we applied transfer learning to train the new model that fits the new HepG2 data. Owing to the pretrained base model, our new model quickly adapted to the new HepG2 cells despite the limited training data available. In practice, we collected stiffness data from only 46 samples under 15 conditions for HepG2 compared to the 104 samples across 22 conditions in the 293T dataset.

After evaluating the model using LOO cross-validation, we observed trends similar to those found in the 293T evaluations. The transfer learning model, when trained with the optimal training hyperparameters, outperformed all other models. It achieved an average testing error of less than 300 Pa, which was less than the measurement standard deviation (Fig. 4b). The average testing errors and scores across other evaluation metrics consistently confirmed that the transfer learning model performed the best (Table 2). Notably, even when the same NN architecture was trained from scratch, it could not match the performance of the transfer learning model. This is likely because the transfer learning model had prior knowledge from the similar 293T dataset, which allowed it to make more realistic assumptions about unknown conditions, especially given the absence of certain critical edge conditions.

**Fig. 5:**
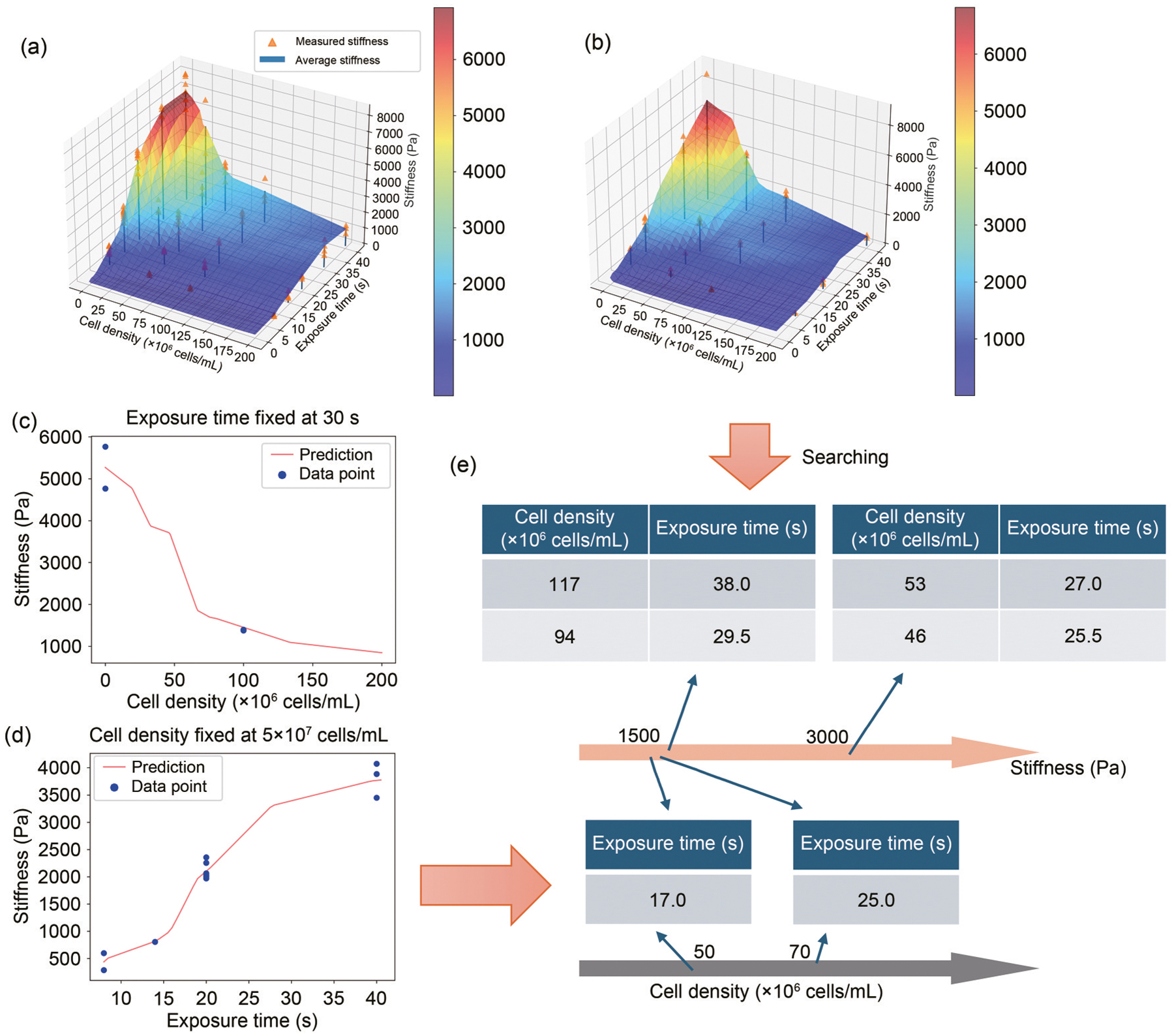
NN prediction results after training with the experimental data. (a) NN model-predicted stiffness values forming a color-gradient surface projected on top of the original measurement data, with 293T cells in the material. Both the color gradient and the *z*-axis values represent the corresponding predicted stiffness values in Pa. (b) NN model-predicted stiffness values for HepG2 cells in the material. (c) Plot of a slice of the predicted stiffness slope along the cell density gradient direction while fixing the exposure time. (d) Plot of a slice of the predicted stiffness slope along the exposure time gradient direction while fixing the cell density. (e) Demonstration of searching the printing parameters from the NN predictions given any target stiffness from the available range

With these optimized models, we modulated the stiffness of future printed scaffolds. With either the HepG2 sample stiffness predictions (Fig. 5b) or the previous 293T sample stiffness predictions (Fig. 5a), we successfully identified combinations of cell density and light exposure time to achieve any targeted stiffness (Fig. 5e). We were also able to fix one parameter, such as exposure time, and determine the corresponding cell density required to achieve a specified stiffness (Fig. 5c). Alternatively, we could fix the cell density and identify the light exposure time necessary to reach a desired stiffness (Fig. 5d). The predicted stiffness ranged from 0.2 to 6 kPa, matching the stiffness of normal liver tissue, typically under 6 kPa [48, 49]. In addition, the predicted stiffness of 3 kPa could be achieved using a cell density of around 5×10^7^ cells/mL, a value that is closer to in vivo cell densities compared to traditional bioprinting methods. This stiffness-controlled bioprinting is especially useful in bioengineering applications, allowing for stiffness adjustments in cell-laden samples, which is currently not feasible without a tedious trial-and-error process consuming excessive quantities of materials and cells. With the help of our NN model, we could now truly control the stiffness of different cell-laden samples. This could further extend to multicell printing, given that we could control the stiffness of the individual cell region utilizing the multimaterial printing function of modern DLP printers.

## 4 Discussion

Our study demonstrated the use of ML and transfer learning for predicting the scaffold stiffness of cell-laden HCD bioprinting using two different cell types, 293T and HepG2. We successfully showed how accurate prediction and modulation of HCD bioprinting could guide the intelligent manufacturing of native tissue mimicking cell-laden scaffolds with designated stiffness. The transfer learning method also highlighted a path for applying our method across distinct cell types, significantly reducing the need for extensive sample collection when training ML models. Here, we will discuss certain experimental design choices made during our study and outline possibilities for future exploration.

In our study, we consistently used a cylindrical sample with a 500 μm diameter and 500 μm height. This printing method, using PDMS spacers to control sample thickness, was widely used in constructing cell-patterned models for investigating cellular activities in 3D cultures [50–52]. For HCD samples, diffusion limitations between 200 and 300 μm can significantly hinder cell viability if the sample is too thick [53]. Therefore, the thin slab samples were more suitable for cellular models. Additionally, compression tests required an aspect ratio close to 1 between sample height and diameter to prevent buckling. As a result, our sample size met the requirements for both general 3D cell culture and mechanical measurement. The stiffness of the samples with different thicknesses could be investigated in the future, and this process could be facilitated by transfer learning.

When considering adjustable parameters in the bioprinting system that could affect scaffold stiffness, we selected cell density and exposure time as input features. This decision focused on the most important factors while minimizing the data requirements for training the ML models. Although other parameters of the bioink composition or printing setting were commonly adjusted in light-based printing, they all affected free-radical concentration during the photopolymerization process. The rationale behind our feature selection was based on three key considerations. First, we sought to model the effects of the cells on the scaffold stiffness. Second, we included easily tunable parameters for manipulating stiffness. Third, we sought to minimize the number of input features to simplify ML model training and reduce data requirements. Cell density and exposure time adequately satisfied these criteria. Material-related features like photoinitiator concentration or prepolymer concentration were excluded as they are either unrelated to cells or not easily adjustable during the printing process. Other DLP printing parameters, like light intensity and mask grayscale intensity, serve a similar role to exposure time by controlling the overall degree of light exposure dosage. Therefore, to reduce training data requirements, we did not include those similar features. The ML model could also be utilized to investigate the influence of these parameters, provided that a sufficient number of stiffness data points are collected. The stiffness obtained and predicted was relatively soft owing to the high cell density. To mimic stiffer tissues, such as liver fibrosis, different bioink compositions or light exposures could be considered. The ML method would still be applicable under these new settings.

Although these features were not selected in the ML model, they were carefully controlled and fixed in all our experiments. Therefore, unlike certain defect detection tasks where uncontrolled variables can affect results, excluding those controlled parameters from our input features did not affect our model’s predictive accuracy. Including those parameters as additional input features and essentially varying them to create more complex input conditions would increase the complexity of the problem, necessitating the collection of more data for ML model training. Expanding the input features to broaden the range of stiffness control remains an intriguing goal. However, this would come at the cost of requiring higher-quality data. We expect that transfer learning will play an important role in training such a complicated problem.

Transfer learning is widely recognized as effective when applied to tasks with a similar scope or knowledge base. In our study, we applied transfer learning on closely related problems, specifically predicting stiffness using the same bioprinter setup and prepolymer material, with the only difference being the cell type. We empirically showed that transfer learning indeed captured the pretrained knowledge from the 293T cell-included bioink and improved the prediction capability of the HepG2 cell case. We assumed that the model effectively learned the distinctions introduced by varying cell types while transferring knowledge about the unchanged factors. Therefore, we propose that the same transfer learning method should still be effective when varying the printer type or material composition, or including additional cell types. We also foresee a potential limitation that the effectiveness of transfer learning may be reduced as task discrepancies increase. This challenge should require robust data collection.

Predicting and controlling the stiffness of more complex 3D structures would be pivotal for tissue engineering applications. Although high-resolution HCD samples with channels can be produced with DLP printing [12], the methods available for mechanical measurement of these complex 3D structures remain restrictive. Techniques like nanoindentation can be applied to more complex surfaces but measure only local stiffness, which may not accurately represent the environment of encapsulated cells. Future work should focus on translating the current ML result from a standard unit to complex structures. For instance, larger, complex 3D shapes could be sectioned into smaller, standardized pieces for mechanical testing. Nevertheless, our work provided a novel solution utilizing ML to predict the stiffness of HCD samples from limited input data. We also demonstrated the effectiveness of transfer learning in adapting the model to accommodate new cell types. This approach could be extended to other factors for 3D printing, including more complex 3D structure design.

## 5 Conclusions

In conclusion, we demonstrated that our NN model, trained on measurement data from bioprinted samples, effectively predicted the stiffness of cell-laden scaffolds. We collected comprehensive stiffness data from the bioprinted GelMA samples encapsulating 293T cells and more limited data from samples encapsulating HepG2 cells. The NN model was carefully chosen and trained using the 293T cell data, delivering exceptional predictive performance that surpassed other ML models. Various ML techniques such as hyperparameter tuning, early stopping, model weight regularization, dropout regularization, and LOO cross-validation were used to ensure that the model avoided overfitting the data and maintained strong generalizability.

Furthermore, we demonstrated that our method could easily adapt to new cell types with limited data by applying transfer learning, as exemplified by our work with HepG2 samples. By collecting fewer than half of the sample measurements, we demonstrated that the properly trained NN model with transfer learning outperformed all comparison models, including the NN model trained from scratch. These findings establish our method as a viable and cost-effective solution for precision bioprinting, enabling scalable stiffness control at low cost.

For future research, we anticipate significant potential for expanding this approach. With the successful integration of IDX to match the bioink’s refractive index offering good viability and flexibility, we expect that our method could be applied to include more complicated input features. Extension to other bioprinters, material compositions, and cell types could also broaden its applicability. With more accurate stiffness measurement techniques, we could potentially extend our method to complex 3D structures and biomimetics. In addition, beyond numerical measurements such as stiffness, the ML model can be a viable option for predicting other quantitative outcomes, such as biomarker expression. To facilitate scaling-up, strategies such as automating data collection and integrating adaptive learning systems that improve with increased data without manual intervention might be employed. Therefore, we could train more robust models and extend them to universal large models that could include multiple cell types and solve multiple tasks simultaneously, for broader adoption in clinical and industrial settings.

## Supplementary Material

supplement

The online version contains supplementary material available at https://doi.org/10.1631/bdm.2400454.

## Figures and Tables

**Table 1:** LOO cross-validation testing evaluations on the 293T data

Method	RMSE	MAPE	*R* ^2^
Linear least square regression	1318	209%	0.57
Linear quadratic regression	971	124%	0.77
SVM	1030	179%	0.74
RF	1007	62%	0.75
1-Layer NN	1019	62%	0.74
Full NN	**726**	**53%**	**0.87**

The best results are in bold

**Table 2:** LOO cross-validation testing evaluations on the HepG2 data

Method	RMSE	MAPE	*R* ^2^
Linear least square regression	1371	158%	0.48
Linear quadratic regression	788	72%	0.83
SVM	1139	200%	0.64
RF	1169	120%	0.62
1-Layer NN	716	65%	0.86
Full NN	684	73%	0.87
Full NN TL 1E-4	549	24%	0.92
Full NN TL 1E-5	**501**	**22%**	**0.93**

The best results are in bold
