# Supplementary material for: Machine learning-assisted stiffness prediction in high-cell-density bioprinting
Source: Biodes Manuf. Author manuscript; Available in PMC 2026 Feb 28. (PMC12948305; doi:10.1631/bdm.2400454)
Supplement: supplement [file NIHMS2149224-supplement-supplement.docx]

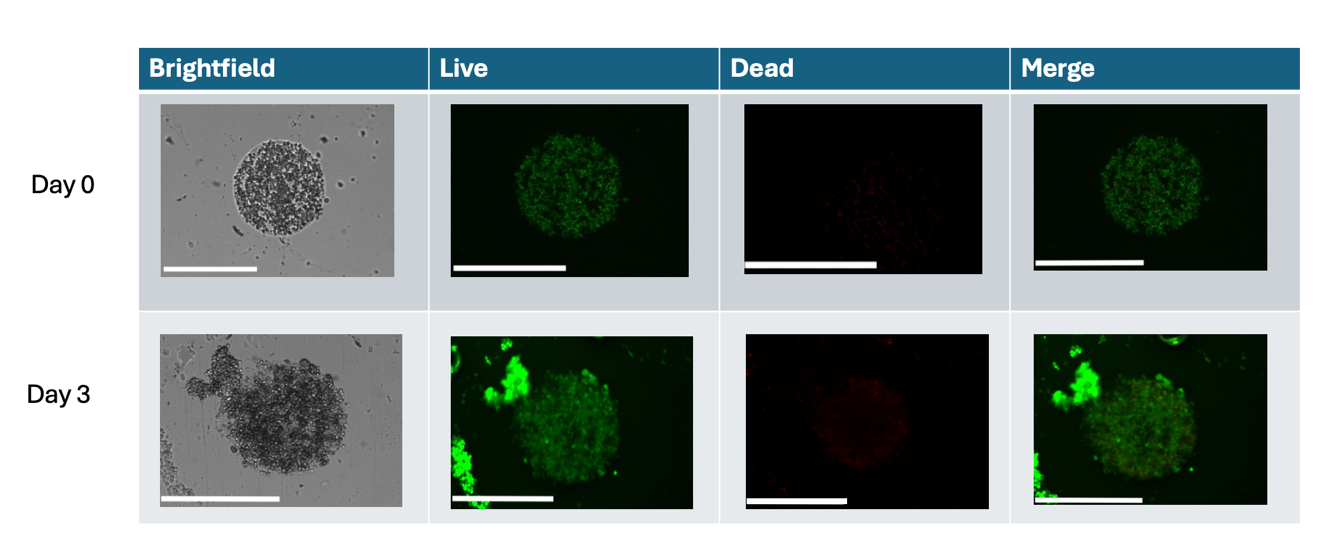


**Fig. S1** Live/Dead staining on high cell density scaffolds printed at 0.2B/mL cell density of 293T cells with 14 second exposure time. The top row represents D0, where the live/dead staining was conducted directly after printing. The bottom row represents a live/dead staining conducted after three days of culture. The scale bars represent 500um


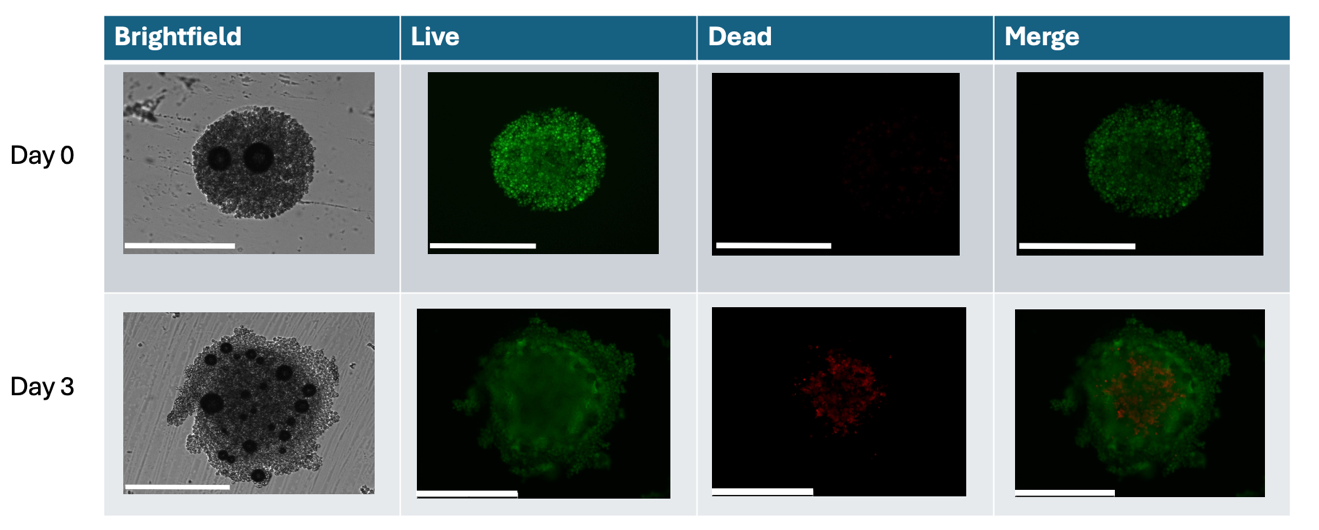


**Fig. S2** Live/Dead staining on high cell density scaffolds printed at 0.2B/mL cell density of 293T cells with 40 second exposure time. The top row represents D0, where the live/dead staining was conducted directly after printing. The bottom row represents a live/dead staining conducted after three days of culture. The scale bars represent 500um


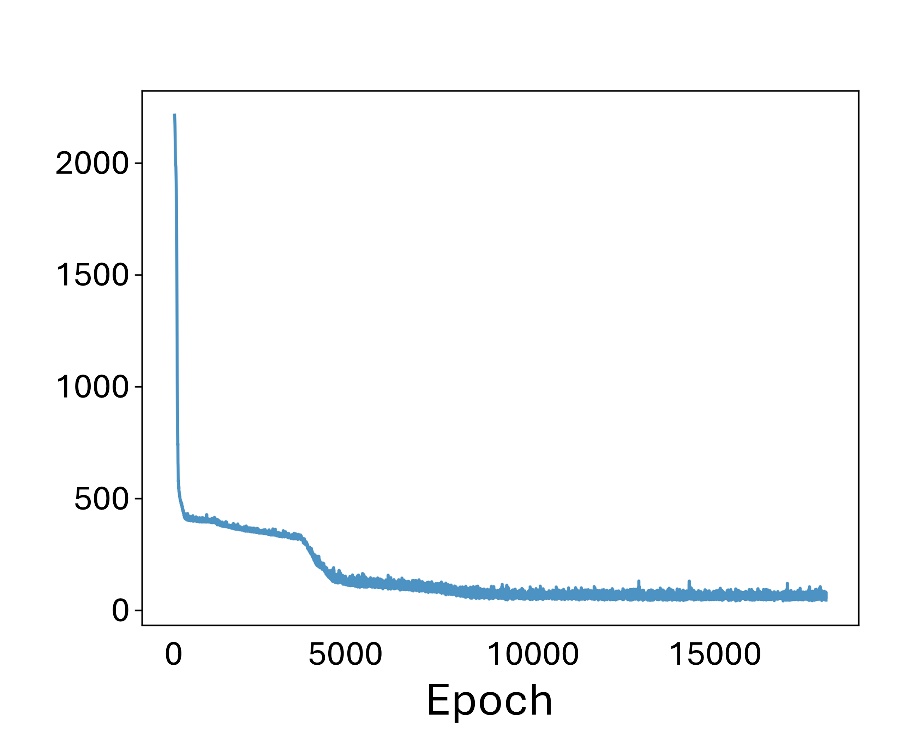


**Fig. S3** A sample training loss plot showing the descending loss during the neural network backpropagation training process.
